# Deep Reinforcement Learning-Based End-to-End Control for UAV Dynamic Target Tracking

**DOI:** 10.3390/biomimetics7040197

**Published:** 2022-11-11

**Authors:** Jiang Zhao, Han Liu, Jiaming Sun, Kun Wu, Zhihao Cai, Yan Ma, Yingxun Wang

**Affiliations:** 1 School of Automation Science and Electrical Engineering, Beihang University, Beijing 100191, China; 2 Flying College, Beihang University, Beijing 100191, China; 3 Science and Technology on Information Systems Engineering Laboratory, Beijing Institute of Control & Electronics Technology, Beijing 100038, China; 4 Institute of Unmanned System, Beihang University, Beijing 100191, China

**Keywords:** unmanned aerial vehicle (UAV), dynamic target, tracking control, deep reinforcement learning (DRL), end-to-end control, neural network

## Abstract

Uncertainty of target motion, limited perception ability of onboard cameras, and constrained control have brought new challenges to unmanned aerial vehicle (UAV) dynamic target tracking control. In virtue of the powerful fitting ability and learning ability of the neural network, this paper proposes a new deep reinforcement learning (DRL)-based end-to-end control method for UAV dynamic target tracking. Firstly, a DRL-based framework using onboard camera image is established, which simplifies the traditional modularization paradigm. Secondly, neural network architecture, reward functions, and soft actor-critic (SAC)-based speed command perception algorithm are designed to train the policy network. The output of the policy network is denormalized and directly used as speed control command, which realizes the UAV dynamic target tracking. Finally, the feasibility of the proposed end-to-end control method is demonstrated by numerical simulation. The results show that the proposed DRL-based framework is feasible to simplify the traditional modularization paradigm. The UAV can track the dynamic target with rapidly changing of speed and direction.

## 1. Introduction

With the continuous improvement of its autonomous intelligence level, unmanned aerial vehicle (UAV) is widely used in the civilian field such as aerial photography, agricultural detection [1,2,3,4,5] and in the military field such as aerial reconnaissance, monitoring [6,7,8]. As a hot and difficult issue, UAV dynamic target tracking technology needs to be solved urgently [9,10,11,12,13,14]. UAV target tracking control needs to realize the whole process closed-loop of sensing control, which has strong systematic and multidisciplinary characteristics. As for the dynamic target tracking task, the movement form of the target is constantly changing, and the randomness, diversity and complexity presented pose a great challenge to the perception and control system of UAV. Due to the lack of prior knowledge of the motion pattern of the tracked target, how to ensure the UAV respond accurately and quickly to the change of the target uncertainty has become an urgent problem to be solved.

Vision-based target tracking control methods can be divided into two categories: traditional target tracking methods [15,16,17,18,19,20] and learning-based target tracking methods [13,21,22,23,24,25,26]. The traditional vision-based UAV target tracking control scheme usually detects the target based on the color, shape and other characteristics, uses the vision-tracking algorithm to estimate the target state according to the image feature points, and then designs the corresponding control law to generate control instructions. The vision-based UAV target tracking control system constructed by [15] is composed of color target detection and tracking algorithm, Kalman filter relative position estimation algorithm and nonlinear controller. Chakrabarty et al. [16] adopts clustering of static-adaptive correspondences for deformable object tracking (CMT) algorithm [17] to realize the tracking of the target on the image plane, which overcomes the problem of poor effect of open tracking-learning-detection (TLD) algorithm in dealing with object deformation. This method has better robustness to target deformation and temporary occlusion. Based on the optimization of hardware, Greatwood et al. [18] designed a ground target tracking controller which can make full use of the parallel characteristics of images and run on the onboard computer efficiently and in real time, and realized the real-time tracking of quadrotor to the ground vehicle target. Diego et al. [19] introduced a Haar feature classifier to realize the recognition of human targets, and combined with the position of the target in the image and Kalman filter algorithm to realize the position tracking and prediction of moving targets. Petersen et al. [20] proposed a UAV target tracking control architecture composed of a vision front-end, a tracking back-end, a selector and a controller. Based on the improved random sampling consensus algorithm, the feature points between adjacent image frames are converted to obtain a moving target tracker, and the final tracked target is determined after screening by the selector.

For the learning-based methods, the UAV target tracking control scheme inspired by the neural network takes the image as the input and directly outputs the action command through the neural network. Kassab et al. [21] realized a target tracking system through two deep neural networks with the aid of image-based visual servo [22]. The proximity network estimates the relative distance between the UAV and the target based on the results of visual tracking, and the tracking network is used to control the relative azimuth between the UAV and the target. Bhagat [13] proposed an algorithm based on deep reinforcement learning (DRL), which takes the position of UAV, target and obstacles in the environment as input, and selects one action of UAV moving in six directions as output. Li [23] proposed a hierarchical network structure that integrates the perception layer and the control layer into a convolutional neural network to realize autonomous tracking of UAV to human. The input of the network is the monocular image and the state information of the UAV. The output includes the four-dimensional motion vector of the three-axis position offset and the heading angle offset. Zhang [24] proposed a coarse-to-fine scheme with DRL to address the aspect ratio variation in UAV tracking. Zhao [25] proposed an end-to-end cooperative multi-agent reinforcement learning (MARL) scheme, in which the UAV can make intelligent flight decisions for cooperative target tracking according to the past and current state of the target. Xu [26] proposed Multiple Pools Twin Delay Deep Deterministic Policy Gradient (MPTD3) algorithm to complete UAV autonomous obstacle avoidance and target tracking tasks, there are often some problems such as slow convergence speed and low success rate. When the target speed changes rapidly, UAV needs to make timely and accurate response to the change of target motion. With the strong fitting ability and learning ability of neural network, combined with many advantages of DRL, this paper focuses on the research of UAV dynamic target tracking control method based on end-to-end learning.

The main contribution of this paper can be summarized as follows:

1. This paper proposes a DRL-based end-to-end control framework of UAV dynamic target tracking, which simplifies the traditional modularization paradigm by establishing an integrated neural network. This method can achieve dynamic target tracking using the policy obtained from the task training of flying towards a fixed target.
2. Neural network architecture, reward functions, and SAC-based speed command perception are designed to train the policy network for UAV dynamic target tracking. The trained policy network can use the input image to obtain the speed control command as an output, which realizes the UAV dynamic target tracking based on speed command perception.
3. The numerical results show that the proposed framework for simplifying the traditional modularization paradigm is feasible and the end-to-end control method allows the UAV to track the dynamic target with rapidly changing of speed and direction.

The remainder of this paper is organized as follows. In Section 2, the problem formulation is stated, and the preliminaries are introduced. In Section 3, the UAV dynamic target tracking control method is proposed in detail, including the framework, neural network architecture, reward functions and SAC-based speed command perception algorithm. In Section 4, numerical results and discussions are presented. Section 5 summarizes the contribution of this paper and presents future work.

## 2. Preliminaries

### 2.1. Problem Formulation

**Figure 1:**
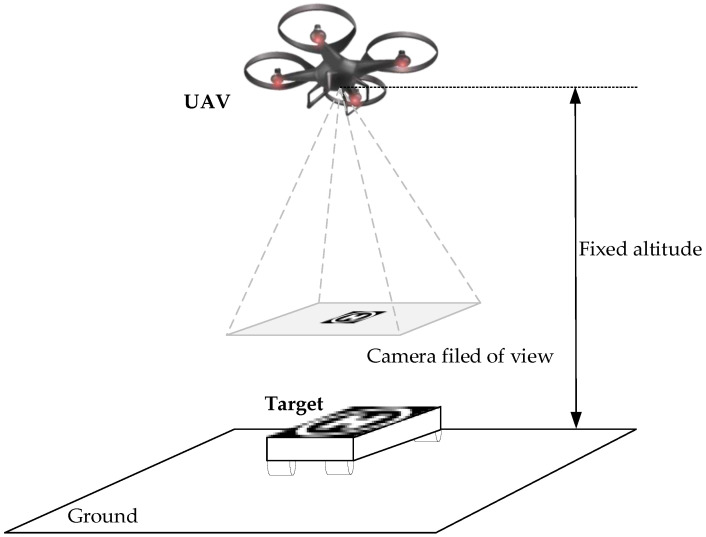
UAV dynamic target tracking problem.

For target tracking problem, the UAV only has prior knowledge about the visual features of the target, but the target motion model is unknown. As Figure 1 shown, the UAV perceives the target and the environment in a limited field of view only through the down-looking monocular camera firmly attached to the bottom of the body. It needs to rely entirely on onboard sensors and onboard computers to process the perception information and generate corresponding control instructions, so that the UAV can track dynamic targets continuously and steadily. To achieve this, an end-to-end control method is proposed to train the UAV to calculate the speed control commands according to the camera image.

### 2.2. UAV Model

**Figure 2:**
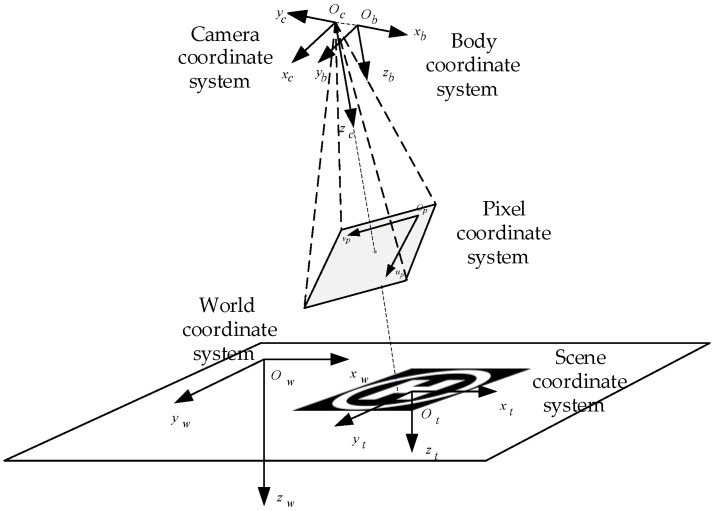
The definition of coordinate system.

As shown in Figure 2, the coordinate systems including the camera coordinate system, body coordinate system, pixel coordinate system, world coordinate system and scene coordinate system.

**Figure 3:**
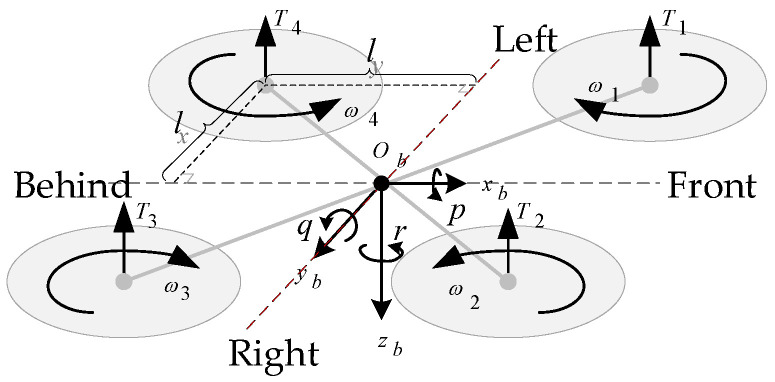
Coordinate systems and forces/moments acting on the UAV.

The UAV studied in this paper is X-configuration quadrotor, and it is symmetrically equipped with four motors, whose rotation drives the rotation of the rotator to generate pull to power the UAV. Figure 3 shows the body coordinate system and forces/moments acting on the UAV.

Assuming that the UAV is a rigid body, it is only subject to gravity in the $O_{w}z_{w}$ direction and lift in the $O_{b}z_{b}$ negative direction. The position and attitude dynamic models of the UAV are shown in Equations (1) and (2) [27].

(1) $$m{\dot{v}}^{w}=\left[\begin{array}{c} 0\\ 0\\ mg \end{array}\right]+R_{b}^{w}\left[\begin{array}{c} 0\\ 0\\ -(T_{1}+T_{2}+T_{3}+T_{4}) \end{array}\right]$$

where $v^{w}={\left[v_{x}^{w},v_{y}^{w},v_{z}^{w}\right]}^{T}$ represents the velocity of the UAV, *m* represents the mass of the UAV, $R_{b}^{w}$ represents the rotation matrix from body coordinate system to world coordinate system, $T_{i}(i=1,2,3,4)$ represents the force generated by the *i*-th motor [28].

(2) $$J\cdot {\dot{\omega }}^{b}=M$$

where *J* represents the inertia of the UAV, M represents total moment of the UAV.

In the process of the UAV tracking the target, it is assumed that the UAV flies at a fixed altitude and its position is recorded as ${\left(X_{UAV}^{w},Y_{UAV}^{w},Z_{UAV}^{w}\right)}^{T}$. The kinematic model of the UAV is shown in Equation (3).

(3) $$\left\{ \begin{array}{l} \left[\begin{array}{c} {\dot{X}}_{UAV}^{w}\\ {\dot{Y}}_{UAV}^{w}\\ {\dot{Z}}_{UAV}^{w} \end{array}\right]=\left[\begin{array}{c} v_{x}^{w}\\ v_{y}^{w}\\ 0 \end{array}\right]\\ \left[\begin{array}{c} \dot{\phi }\\ \dot{\theta }\\ \dot{\psi } \end{array}\right]=\left[\begin{array}{ccc} 1 & \tan\theta \sin\phi & \tan\theta \cos\phi \\ 0 & \cos\phi & -\sin\phi \\ 0 & \sin\phi /\cos\theta & \cos\phi /\cos\theta \end{array}\right]\left[\begin{array}{c} p\\ q\\ r \end{array}\right] \end{array}\right.$$

The target moves in the $O_{w}x_{w}y_{w}$ plane, and its position is recorded as ${\left(X_{T}^{w},Y_{T}^{w},0\right)}^{T}$. The kinematic model of the target is shown in Equation (4).

(4) $$\left[\begin{array}{c} {\dot{X}}_{T}^{w}\\ {\dot{Y}}_{T}^{w} \end{array}\right]=\left[\begin{array}{c} v_{{}_{Tx}}^{w}\\ v_{{}_{Ty}}^{w} \end{array}\right]$$

In order to describe the relative motion relationship between the UAV and the target, the position vector of the target relative to the UAV in the world coordinate system is defined as ${\left(X_{R}^{w},Y_{R}^{w},Z_{R}^{w}\right)}^{T}={\left(X_{T}^{w},Y_{T}^{w},Z_{T}^{w}\right)}^{T}-{\left(X_{UAV}^{w},Y_{UAV}^{w},Z_{UAV}^{w}\right)}^{T}$. The position tracking error between the UAV and the target is defined as the difference between the coordinates of the current position of the UAV and the target.

### 2.3. DRL and SAC

**Figure 4:**
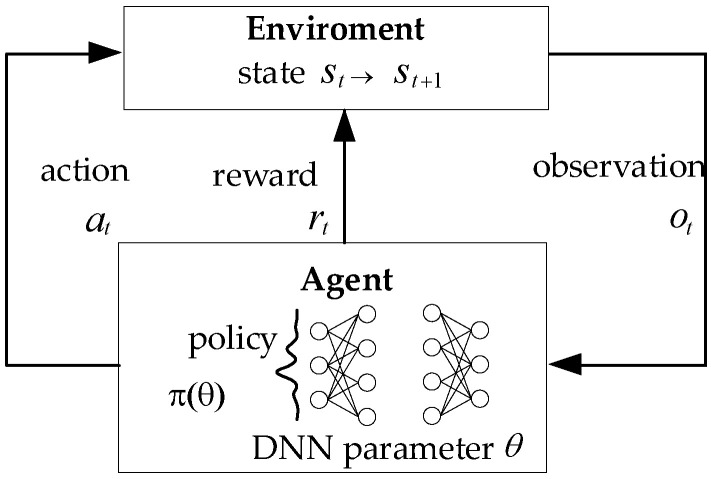
Framework of DRL.

DRL is a cross field of reinforcement learning (RL) and deep neural network. DRL method can perceive complex inputs and make decisions at the same time. Figure 4 shows the basic framework of DRL. This figure can well reflect the interactive characteristics of DRL. At each time step *t*, the agent interacts with the environment once. The agent is in state $s_{t}$ and generates action $a_{t}$ according to the policy $\pi (a_{t}|s_{t};\theta )$ represented by the neural network parameter θ. After the action acts on the environment, the state of the agent will be updated to $s_{t+1}$ according to the dynamic model of the environment $p(s_{t+1},r_{t}|s_{t},a_{t})$. At the same time, the immediate reward $r_{t}$ for obtaining environmental feedback is obtained. Therefore, the DRL problem aims to give a series of interaction processes between agent and the environment, and finds the optimal policy to maximize the return $R_{t}$.

**Figure 5:**
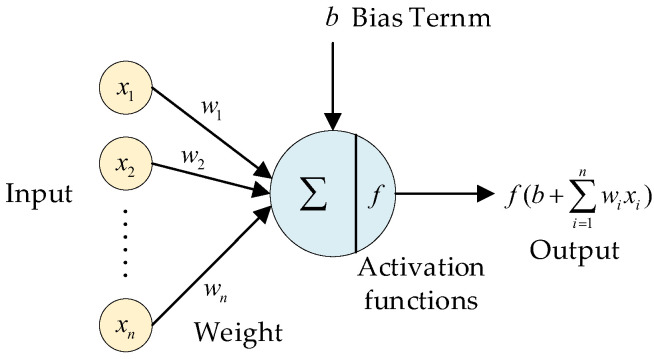
A single neuron.

Neural network is a mathematical model that simulates the structure of biological neural network and performs distributed parallel information processing. It can adaptively change its own structural parameters based on external information, and store information with the help of parameters such as weight and bias term of each layer. The smallest unit node that constitutes the neural network model is “neuron”, as shown in Figure 5. The output of a neuron in an episode is the result of the activation function after the addition of the weighted sum of the input data and the bias term. The activation function provides the nonlinear expression ability for the neural network, which is differentiable and monotonic [29].

The SAC algorithm is an actor-critic DRL algorithm that introduces maximum entropy, in which the actor generates a random policy. The goal of this algorithm is to maximize the cumulative reward regularized by entropy instead of just the cumulative reward. SAC algorithm can increase the randomness of action selection, encourage the agent to explore more in the training process, and thereby speed up subsequent learning to prevent the policy from prematurely converging to a local optimum. Some practical experiments show that SAC algorithm has higher learning efficiency than RL algorithm with traditional objective function.

The basic principle of SAC algorithm is briefly described below. π(a|s;θ) represents the actor network with parameter θ, Q(s,a;ϕ) represents Q-network with parameter ϕ. V(s;ψ) and $\bar{V}(s;\psi )$, respectively, represent the behavior value network and the corresponding target value network. Q-network and V-network together form a critic for the evaluation of the actor network. In each iteration, the agent first interacts with the environment based on the current policy to generate new data and stores it in the experience pool, and then randomly samples from the experience replay buffer and updates the actor, critic and the corresponding target network. Through derivation, the loss function of the V-network is [30]:

(5) $$J_{V}(\psi )=E_{s_{t}\sim D}[\frac{1}{2}{\left(V_{\pi }(s_{t};\psi )-E_{a_{t}\sim \pi (\theta )}[Q_{\pi }(s_{t},a_{t};\phi )-\alpha \log\pi (a_{t}|s_{t};\theta )]\right)}^{2}]$$

where α is the temperature parameter that determines the relative importance of the entropy term versus the reward, and thus controls the stochasticity of the optimal policy. The gradient of V-network is:

(6) $${\hat{\nabla }}_{\psi }J_{V}(\psi )=\nabla_{\psi }V(s_{t};\psi )(V(s_{t};\psi )-Q_{\pi }(s_{t},a_{t};\phi )+\alpha \log\pi (a_{t}|s_{t};\theta ))$$

the loss function of the Q-network is:

(7) $$J_{Q}(\phi )=E_{(s_{t},a_{t})\sim D}[\frac{1}{2}{\left(Q_{\pi }^{}(s_{t},a_{t};\phi )-{\hat{Q}}_{\pi }^{}(s_{t},a_{t})\right)}^{2}]$$

where, ${\hat{Q}}_{\pi }^{}(s_{t},a_{t})=r(s_{t},a_{t})+\gamma E_{s_{t+1}\sim p}[V_{}^{}(s_{t+1};\overset{-}{\psi })]$. γ is the discount factor to ensure that the sum of expected rewards (and entropy) is finite. Then the gradient of Q-network is:

(8) $${\hat{\nabla }}_{\phi }J_{Q}(\phi )=\nabla_{\phi }Q_{\pi }^{}(s_{t},a_{t};\phi )(Q_{\pi }^{}(s_{t},a_{t};\phi )-r(s_{t},a_{t})-\gamma V(s_{t+1};\overset{-}{\psi }))$$

Since actor network generates a random policy, under the setting of continuous action space, re-parameterization is introduced to update the policy to reduce the variance of policy gradient estimation. The policy π(θ) is expressed as a function that uses the state *s* and the noise vector ε subject to the normal distribution as input to output the action *a*, that is a=f(s,ε;θ). This process can also be regarded as action sampling from the normal distribution determined by the output of the policy network, and then the loss function of the actor network can be obtained as [31]:

(9) $$J_{\pi }(\theta )=E_{s_{t}\sim D,\varepsilon_{t}\sim N}[\log\pi (f(s_{t},\varepsilon_{t};\theta )|s_{t};\theta )-Q_{\pi }^{}(s_{t},f(s_{t},\varepsilon_{t};\theta );\phi )]$$

## 3. End-to-End Control for UAV Dynamic Target Tracking

### 3.1. Framework

**Figure 6:**
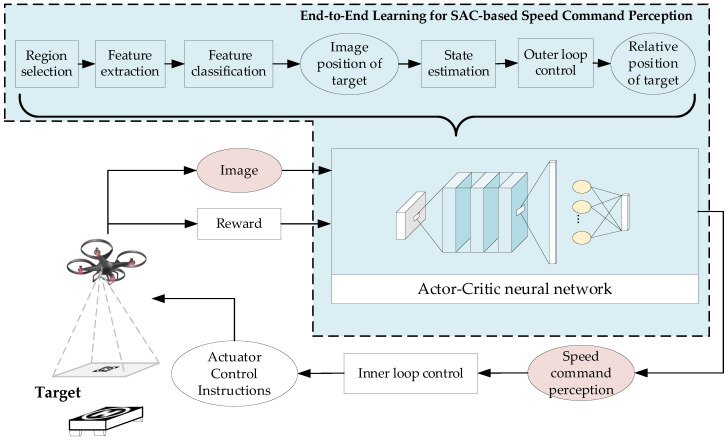
Framework of UAV dynamic target tracking control based on DRL.

Based on DRL, we design the end-to-end control method of directly outputting speed control commands from the original images during the interaction between the UAV target tracking agent and the simulation environment. End-to-end control method can directly learn the corresponding control strategy based on high-dimensional sensor input information by establishing a certain structure of depth neural network between the sensing end and the control end, and replacing the manually designed features with automatically extracted hierarchical depth features. The speed control commands can be directly obtained as the input of the subsequent controller through the inverse normalization processing of the network output, to realize the perception control closed loop of the UAV dynamic target tracking. The framework of UAV dynamic target tracking control based on DRL is shown as Figure 6.

#### 3.1.1. Markov Decision Process for Target Tracking

Markov decision process is the most important mathematical model in RL. Therefore, the dynamic target tracking control problem of UAV is first described by Markov decision process, with the emphasis on the definition of its state space and action space.

**Figure 7:**
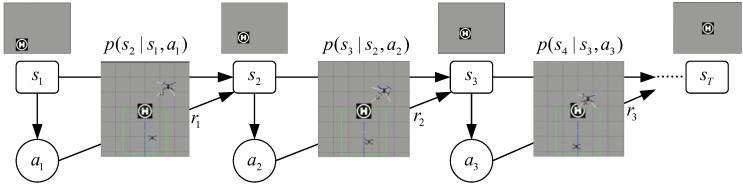
Markov decision process for UAV dynamic target tracking.

It can be seen from the analysis of the UAV dynamic target tracking task that the camera image and the state of the UAV at the next moment only depend on the control command generated and executed according to the current image. Therefore, the camera image of the UAV is regarded as an observable state $s_{t}$, and the control command is regarded as an action $a_{t}$. The alternation between the two will form a set of state action sequences in time sequence within a finite time domain, recorded as trajectory $\tau =s_{0},a_{0},\cdots ,s_{t-1},a_{t-1},s_{t},\ldots ,a_{T-1},s_{T}$, where $s_{0}$ is the initial state, and *T* is the termination time of the finite time domain. Figure 7 shows the Markov decision process for UAV dynamic target tracking.

The Markov decision process of UAV target tracking can be described by tuples {S,A,P,R,γ}: S is the state space. Considering that the original image size of the UAV onboard camera is large, the image after size compression and pixel value normalization to [0,1] is defined as the state, therefore, $S=\{s\in {\mathbb{R}}^{120\times 120\times 3}\}$. A is the action space. The actions are defined as the desired speed control commands of the UAV normalized in the horizontal direction, therefore $A=\{a={\left(v_{cmd\_x},v_{cmd\_y}\right)}^{T}|v_{cmd\_x},v_{cmd\_y}\in [-1,1]\}$. P is the state transition function and can be recorded as P:S×A×S→[0,1]. The meaning of this function is the probability $p(s^{\prime }|s,a)$ that the UAV will acquire the image $s^{\prime }$ after sensing the image s and taking action a. R is the reward function and can be recorded as R:S×A→ℝ. γ is discounting factor and can be used to calculate cumulative rewards, γ∈(0,1).

#### 3.1.2. Interactive Environment and Agents

The environment in the DRL problem refers to the sum of various peripheral elements that interact with the agent, including interface functions to achieve interaction and entities such as targets and surrounding scenes. The interface functions used for UAV target tracking task mainly include initialization function, reset function and single step interaction function.

The initialization function is used to declare and initialize the parameters of the environment and some global variables shared by multiple functions, including the altitude of the UAV flying at fixed altitude, the starting position of the target, the boundary of the camera field of view, and the number of interaction steps. The reset function is used to reset the UAV, target and global variables in the environment when the agent triggers the episode termination condition, and returns the image observed by the UAV at the reset position as the initial state of the new episode. The single-step interaction function is used to make the agent interact with the environment once in each training step. The normalized action generated by the agent at the current time is taken as the input, and after the speed control command is restored and limited, it is forwarded to the UAV model for execution. Next, the physical state of the UAV is updated at a certain interval, and the new image in the field of view can be obtained as the new state to which the interaction is transferred. Then, the reward function calculates the single-step reward and determines whether the episode termination conditions are met. Finally, the function returns the normalized new state, the single-step reward, the episode end flag amount, and the related annotation information.

The agent in DRL has the ability of action decision-making and self-renewal, and the core is the policy itself approximated by the deep neural network, that is π:S×A→[0,1], represents the transition probability from state to action. Since the state space of the UAV target tracking control problem is a high-dimensional space, in the design of the policy network, the design idea of multiple feature extraction network is adopted. The multilayer convolutional neural network is selected as the first half of the policy network π(a|s;θ), and a hidden layer with spatial feature extraction function is added before the full connection layer of the second half to enhance the expression of the position information of the target in the image. Considering the stability and convergence effect of neural network training, it is also necessary to normalize the state input of the agent policy.

The use of agents has two modes: training and testing. The agent is designed with actor-critic framework, so it needs to maintain both actor and critic networks during the training process, but only needs to run actor network during the testing process. In the training mode, the agent uses the collected interactive data to iteratively update its policy network parameters according to certain rules. The policy gradually converges to the vicinity of the optimal policy, making the action output generated according to the state input more and more ideal and accurate. In the testing mode, the trained agent policy network is loaded and its parameters and structure are fixed. The agent only needs to perform forward propagation calculation according to the incoming image state $s_{t}$ at each time step to obtain the action output $a_{t}$.

### 3.2. Neural Network Architecture for End-to-End Learning

**Figure 8:**
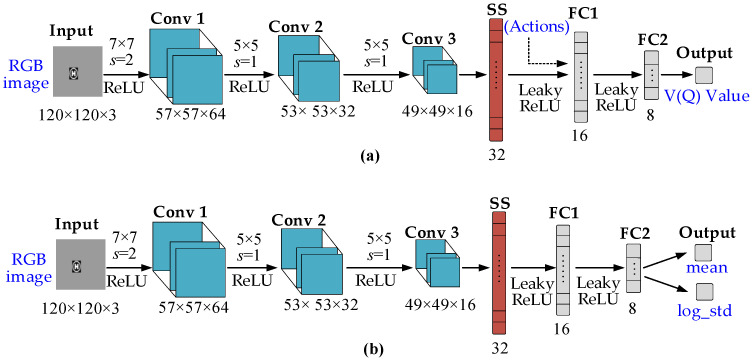
The neural network architecture: (**a**) the architecture of actor-network; (**b**) the architecture of critic-network.

The designed actor network architecture and critic network architecture for end-to-end learning are shown in Figure 8. Their backbone networks are both composed of three convolution layers and spatial index normalization layers.

The actor network has two branches at the last output layer, which are, respectively, used to calculate the mean and logarithmic variance of the generated random action Gaussian distribution. The critic network is behind the backbone network. For the Q- network, the upper layer feature vector should be spliced with the current action vector as the input of the subsequent fully connected layer, and the final output is a scalar, that is, the estimated Q value; while for the V-network, the action vector is not required as an additional input. The feature vector output by the spatial index normalization layer can be used as the input of the subsequent fully connected layer, and the final output is the V value.

### 3.3. Reward Function for Target Tracking

The designed reward function is mainly composed of three items. The first item $r_{1}$ is related to the relative distance between the UAV and the target in the horizontal direction at the current time. The second item $r_{2}$ is related to the action direction calculated by the agent at the current time and the relative orientation between the UAV and the target, and the third item $r_{3}$ is related to the episode termination condition.

The design of $r_{1}$ is to encourage the action of the UAV to approach the target and punish the action of the UAV to move away from the target. $d_{r}$ represents the distance between the UAV and target in the current step; $d_{r\_last}$ represents the distance between the UAV and target in the last step; $d_{\lim}$ represents the limit distance that the UAV can track the target. When $d_{r}=d_{\lim},d_{r\_last}>d_{r}$, we set $r_{1}=0$. The closer the UAV is to the target in the process of approaching the target, the greater the positive reward value obtained; the farther the UAV is from the target in the process of moving away from the target, the greater the absolute value of the negative reward obtained. Since the reward in an episode is the accumulated reward value for a period of time, when the UAV changes from approach the target in the previous step to moving away from the target in the current step, the absolute value of the punishment in the current step is greater than the reward in the previous step. When the UAV approaches the target for several consecutive steps, it is weighted by the number of consecutive steps. $r_{1}$ is calculated as follows:

(10) $$r_{1}=\left\{ \begin{array}{l} (-5d_{r}+5d_{\lim})\cdot n_{approach},\;\mathrm{if}\;d_{r\_last}>d_{r}\\ 5d_{r\_last}-5d_{\lim}-2d_{r},\;\mathrm{if}\;d_{r\_last}<d_{r} \end{array}\right.$$

where approach step $n_{approach}$ is cleared when the UAV is far from the target, and accumulated by 1 when the UAV approaches the target.

The design of $r_{2}$ is to reward and punish the action direction. Firstly, the actual azimuth angle of the target relative to the UAV is calculated based on the current position of the UAV and the target. Secondly, the included angle $\theta_{error}$ between the action direction vector a and the actual relative azimuth direction vector $a_{\theta_{r}}$ is calculated by using the cosine theorem. If the included angle is less than a threshold $\theta_{thresh}$, a positive reward inversely proportional to the included angle is given; otherwise, set this item as negative reward and the greater the included angle, the greater the absolute value of the negative reward. In addition, in order to prevent an excessive value when $\theta_{thresh}$ approaching 0, it is necessary to limit it when $r_{2}$ is a positive reward. $r_{2}$ is calculated as follows:

(11) $$\theta_{error}=\arccos(\frac{a\cdot a_{\theta_{r}}}{\left|a|\cdot |a_{\theta_{r}}\right|}),|a_{\theta_{r}}|=d_{r}$$

(12) r2={12θerror, if θerror<θthresh−2θerror, else

In the design of $r_{3}$, the judgment of episode termination conditions is mainly considered. Assuming three conditions to trigger the termination of the episode: the failure of the episode mission caused by the loss of the target in the field of vision, the success of the episode mission caused by the UAV moving to a certain area directly above the target and meeting certain conditions, and the maximum number of steps reached in the episode. The reward function is only set for the first two conditions in this paper. When the relative distance between the UAV and the target are greater than the geographical boundary $d_{\lim}$ constrained by the camera’s field of vision, it is deemed that the mission of this episode has failed. The UAV is directly given a negative reward $r_{out}$ with a large absolute value and the influence of the other two rewards is shielded. When the horizontal distance between the UAV and the target is less than a certain threshold $d_{r\_thresh}$, it is considered that the UAV has successfully completed the task. At this time, a positive reward $r_{success}$ weighted by the times of consecutive successes $n_{success}$ is added based on the first two rewards. The times of successes $n_{success}$ only counts the number of consecutive steps that meet the UAV’s threshold above the target.

(13) $$r_{3}=\left\{ \begin{array}{l} r_{out},\;\mathrm{if}\;d_{r}>\;d_{\lim}\\ n_{success}\cdot r_{success},\;\mathrm{if}\;d_{r}<d_{r\_thresh}\\ 0,\;\mathrm{else} \end{array}\right.$$

In conclusion, the reward function designed in this paper for the DRL problem of UAV target tracking has the following form:

(14) $$r=\left\{ \begin{array}{l} r_{3},\;\mathrm{if}\;d_{r}>d_{\lim}\\ w_{1}\cdot r_{1}+w_{2}\cdot r_{2}+r_{3},\;\mathrm{else}\; \end{array}\right.$$

where $w_{1},w_{2}$ are the corresponding weight coefficients.

### 3.4. SAC-Based Speed Command Perception

To reduce the deviation of Q value calculation, two Q-networks are maintained during actual training, and the smaller Q value among the outputs of the two Q-networks are used to calculate the loss of the policy network and the V-network [32]. Note that two Q-networks are $Q_{1}(s,a;\phi_{1})$ and $Q_{2}(s,a;\phi_{2})$, respectively, then the loss function of the V-network is:

(15) $$J_{V}(\psi )=E_{s_{t}\sim D}[\frac{1}{2}{\left(V_{\pi }^{}(s_{t};\psi )-E_{a_{t}\sim \pi (\theta )}[\underset{i=1,2}{\min}Q_{i}(s_{t},{\tilde{a}}_{t};\phi_{i})-\alpha \log\pi (a_{t}|s_{t};\theta )]\right)}^{2}]$$

where ${\tilde{a}}_{t}$ is also obtained based on action sampling, that is, ${\tilde{a}}_{t}=f(s_{t},\varepsilon_{t};\theta )$.

Accordingly, the loss function of the policy network is:

(16) $$J_{\pi }(\theta )=E_{s_{t}\sim D,\varepsilon_{t}\sim N}[\log\pi (f(s_{t},\varepsilon_{t};\theta )|s_{t};\theta )-\underset{i=1,2}{\min}Q_{i}(s_{t},f(s_{t},\varepsilon_{t};\theta );\phi_{i})]$$

**Figure 9:**
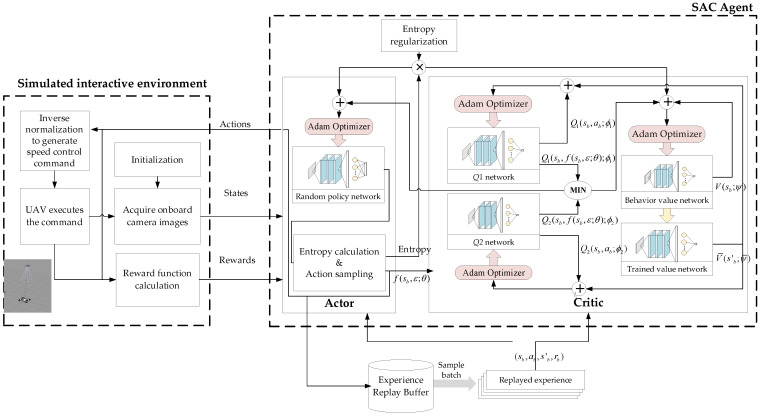
The diagram of the SAC-based speed command perception algorithm implement.

Then, end-to-end learning for speed command perception training framework based on SAC algorithm can be obtained, as shown in Figure 9.

The SAC-based training algorithm is summarized as Algorithm 1. In practical application, the agent only needs to load the neural network model obtained through the above training process, and perform inverse normalization processing on the network output to generate speed control commands for interaction with the environment.
**Algorithm 1.** **SAC-Based Training Algorithm**1.Initialize the learning rate of each network $\lambda_{V},\lambda_{Q},\lambda_{\pi }$;2.Initialize the target network soft update rate τ, entropy regularization weights α;3.Initialize each network parameter $\theta ,\phi ,\psi ,\overset{-}{\psi }$;4.Initialize the replay buffer;5.For each episode:
(1)Initialize the UAV starting position;(2)Reset various parameters in the interactive environment;(3)Receive initial observation of the image state $s_{0}$;(4)For each time step t=1,2,3…:
Take the current state as the input of the current actor network, and generate actions $a_{t}$;Normalize the actions and convert them into speed control commands;Control the UAV with control commands and observe reward $r_{t+1}$ and observe new image state $s_{t+1}$;Store the piece of experience $\left(s_{t},a_{t},s_{t+1},r_{t+1}\right)$ into the replay buffer;Sample replayed experience $\left(s_{b},a_{b},s_{b}^{\prime },r_{b}\right)$ from the replay buffer;Update the behavior value network: $\psi \leftarrow \psi -\lambda_{V}{\hat{\nabla }}_{\psi }J_{V}(\psi )$;Update the Q network: $\phi \leftarrow \phi -\lambda_{Q}{\hat{\nabla }}_{\phi }J_{Q}(\phi )$;Update the policy network: $\theta \leftarrow \theta -\lambda_{\pi }{\hat{\nabla }}_{\theta }J_{\pi }(\theta )$;Update the target value network: $\bar{\psi }\leftarrow \tau \psi +(1-\tau )\bar{\psi }$;If the terminal condition is satisfied, start a new episode. Or, continue for next time step. The end of a time step;The end of an episode;

## 4. Numerical Simulations

In this section, training simulation and three UAV dynamic target tracking simulations with different conditions are executed to test the performance of the trained policy network.

### 4.1. Training Results

In this subsection, the policy network is trained by the proposed method. In order to test the task completion ability of the obtained policy network, 100 random start point tests are conducted using the simulation scenario including the target fixed at (0,0,0) m during the training.

As for the training parameters setting, the size of experience replay buffer is 10,000. The size of experience replay batch is 128. The discounting factor is 0.99. The maximum number of steps each episode is 50. The learning rate of each neural network is 0.0003, as shown in Table 1.

**Figure 10:**
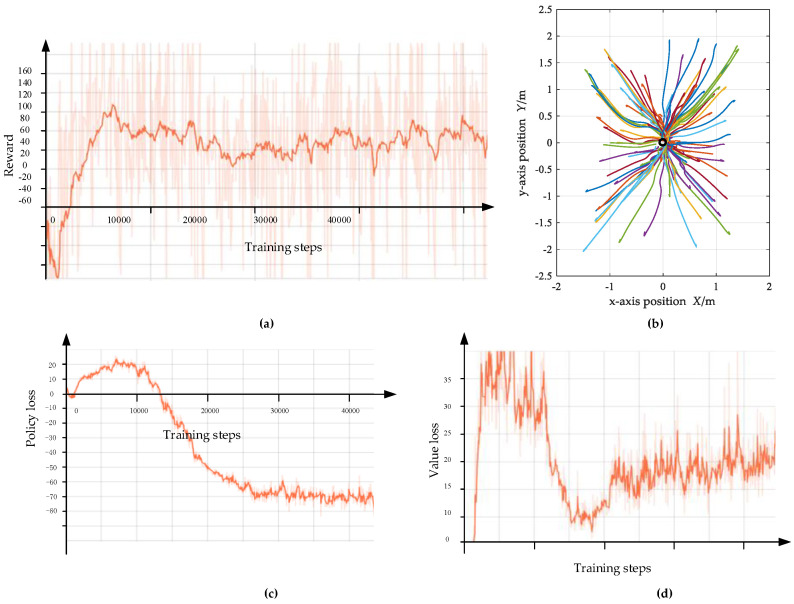
The training results of UAV target tracking. (**a**) The variation curve of episode cumulative reward in training process of speed command perception; (**b**) the position of UAV and target; (**c**) evaluation policy loss result of in training process; (**d**) evaluation value loss results of in training process.

After about 40,000 steps of interaction, the cumulative reward variation curve of the episode is shown in Figure 10a. It can be seen that it rises rapidly at about 5000 steps, and then maintains a large positive level, which indicates that the agent has learned the policy of obtaining a large cumulative reward through interaction with the environment, which reflects the effectiveness of the end-to-end learning process of speed command perception. For DRL agents, the success rate is generally used as an indicator to measure the quality of training results. The trajectories of the UAV during the testing are shown in Figure 10b. The UAV can fly from any starting position to the top of the fixed target, and the mission success rate is 100%. It shows that the obtained policy network is feasible to complete the equivalent task of UAV dynamic target tracking. In the following simulation experiments, the policy network is directly used to track the dynamic target. One of the evaluation loss results of actor and critic network from our many trainings are shown in Figure 10c,d.

### 4.2. Dynamic Target Tracking

In this subsection, the target tracking effect of UAV is tested under three conditions that the target moves along different trajectories, as shown in Table 2. The altitude of UAV fixed altitude flight is set to 5 m.

Detailed description and analysis of the simulation results under each condition are given in the following.

#### 4.2.1. Case 1: Square Trajectory

For the first case, the target starts at (0,0,0) m and moves along a square trajectory with a side length of 8 m. The target makes a uniform linear motion on the side of the square, and when it moves to the vertex of the square, the velocity direction will change by 90° and then continue to move in a uniform linear motion in the new direction. The UAV hovers directly above the target at the start time.

**Figure 11:**
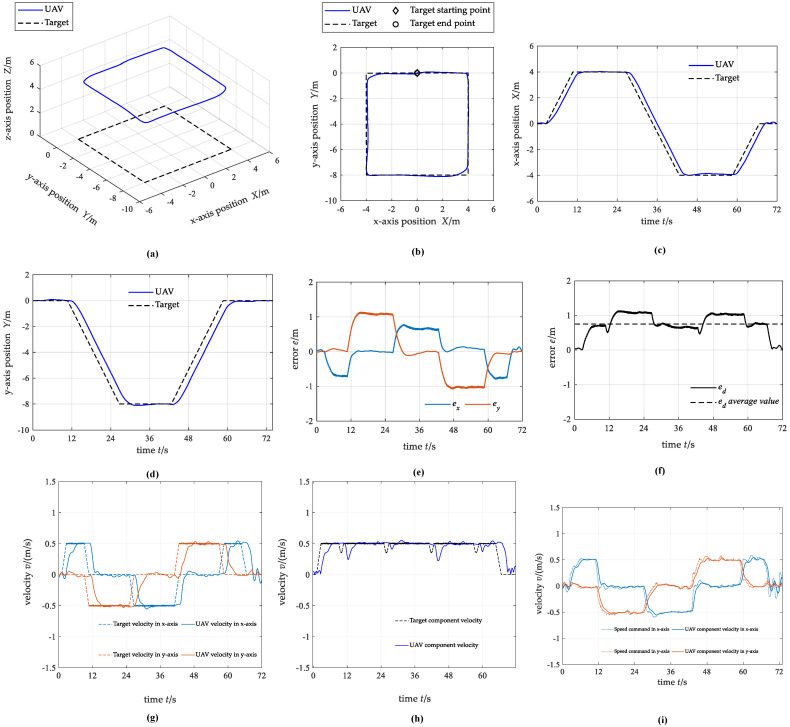
The state curves of UAV and target under case 1. (**a**) 3D trajectory; (**b**) 2D trajectory in the x-y plane; (**c**) *x*-axis position; (**d**) *y*-axis position; (**e**) position tracking error; (**f**) distance tracking error; (**g**) component velocity; (**h**) resultant velocity; (**i**) control commands for the actuators.

Figure 11 shows the simulation testing results. In summary, the UAV can complete the stable tracking of the dynamic target along the square trajectory. The UAV has no overshoot in the process of tracking the target, and the average distance tracking error between the UAV and the target is 0.75 m. The tracking effect of the UAV in the x-axis direction and the y-axis direction is different. The absolute value of the maximum position tracking error in the *x*-axis direction is 0.80 m, but in the y-axis direction is about 1.14m. This situation may be caused by the uneven distribution of interaction data used in training in all directions. The velocity of UAV can keep stable near the target velocity, and rarely exceeds the target velocity, which indicates that the velocity control command directly generated by the neural network is conservative.

#### 4.2.2. Case 2: Polygonal Trajectory

For the second case, the target starts at (0,0,0) m and moves along a polygonal trajectory. Specifically, after the target moves along the straight line y=x for a certain distance, the sudden change of −135° occurs in the velocity direction and moves along the negative direction of the *y*-axis for a certain distance, and then the sudden change of +135° occurs in the velocity direction. After repeating this for several times, the target stops moving. The UAV hovers directly above the target at the start time.

**Figure 12:**
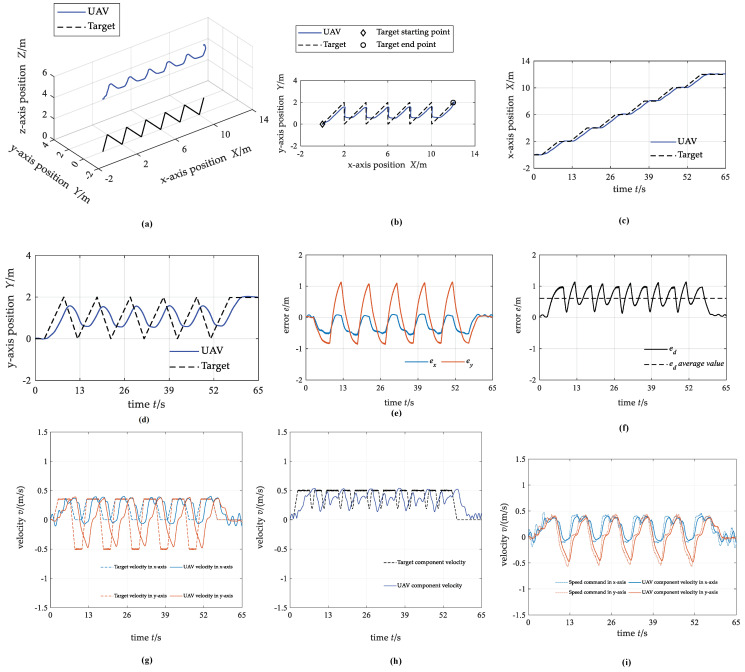
The state curves of UAV and target under case 2. (**a**) 3D trajectory; (**b**) 2D trajectory in the x−y plane; (**c**) x−axis position; (**d**) y−axis position; (**e**) position tracking error; (**f**) distance tracking error; (**g**) component velocity; (**h**) resultant velocity; (**i**) control commands for the actuators.

Figure 12 shows the simulation testing results. The tracking effect of the UAV in the *x*-axis direction where the target motion change is relatively gentle is acceptable and the absolute value of the maximum position tracking error in this direction is about 0.57 m. However, the tracking hysteresis in the *y*-axis direction where the target motion changes greatly is obvious and the absolute value of the maximum position tracking error in the *y*-axis direction is about 1.14 m. The average distance tracking error between UAV and target is about 0.61 m.

#### 4.2.3. Case 3: Curve Trajectory

For the third case, the target starts at (0,0,0) m and moves along a lemniscate curve trajectory. The moving velocity of the target is slower at the place where the curvature of the lemniscate is small and faster at the place where the curvature is large, and generally changes within the range of 0.5 m/s~1.2 m/s. The UAV hovers directly above the target at the start time.

**Figure 13:**
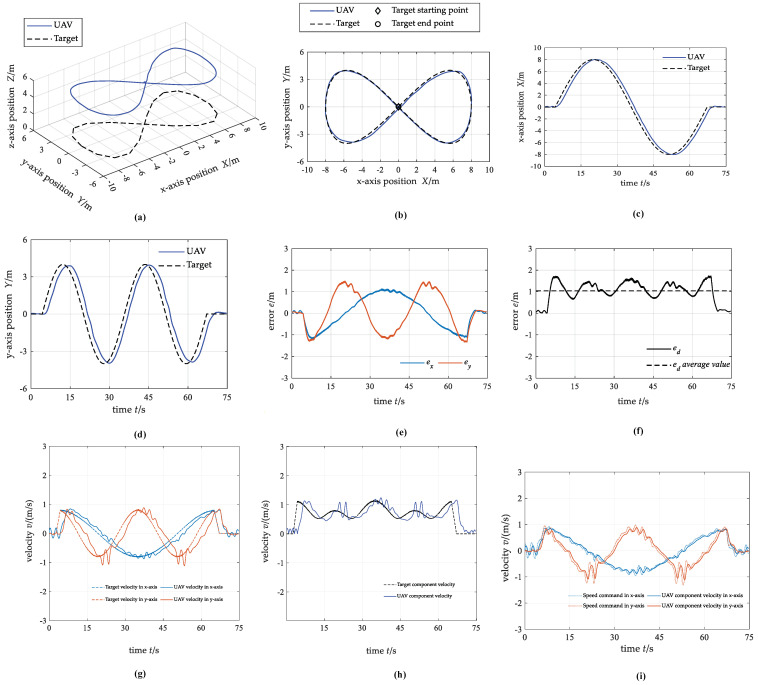
The state curves of UAV and target under case 3. (**a**) 3D trajectory; (**b**) 2D trajectory in the x−y plane; (**c**) x−axis position; (**d**) y−axis position; (**e**) position tracking error; (**f**) distance tracking error; (**g**) component velocity; (**h**) resultant velocity; (**i**) control commands for the actuators.

Figure 13 shows the simulation testing results. In summary, the motion trajectory of UAV and target fit well. The average value of the distance tracking error between the UAV and the target is 1.04 m. The absolute value of the maximum position tracking error in the *x*-axis direction is about 1.18 m, and in the *y*-axis direction is about 1.49 m. The velocity of the UAV fluctuates at the local maximum of the target velocity, which indicates that the tracking is difficult when the target velocity changes greatly.

### 4.3. Discussion

The numerical simulation results under different cases demonstrate the effectiveness of the dynamic target tracking control method of UAV based on DRL. Through three groups of simulation tests, we examine the performance of UAV when tracking target with constant speed, target with sudden change in direction, and target with changing speed and direction.

In the first two groups of simulations, the directions of the target speed changes are 90° and 135°, respectively. In the first simulation, the component speed of UAV can keep 0.5 m/s. While in the second simulation, the component speed of UAV is only 0.33 m/s. Therefore, the greater the change of target speed direction, the more difficult for UAV dynamic target tracking. In the third simulation, the speed and direction of target concurrently change, and the distance error of this simulation is 1.04 m, larger than the other two simulations. All the simulation results show that the UAV can complete the target tracking task under various conditions.

## 5. Conclusions

This paper proposes a new DRL-based end-to-end control method for UAV dynamic target tracking. This method is demonstrated based on several numerical simulation experiments. The UAV can track target with sudden changes of 90° or 135° in direction and target with speed varying from 0.5 m/s to 1.2 m/s. In addition, the SAC-based algorithm can accelerate the subsequent learning speed and prevent the policy from converging to the local optimal value prematurely. The end-to-end control using neural networks can be used for obstacle avoidance, landing control, et al. For future work, the model trained by neural network method will be used in flight experiment to demonstrate the feasibility of the end-to-end control method. A comparison between the proposed method with the traditional methods will also be performed.

## Figures and Tables

**Table 1:** The parameters of training simulation.

Training Parameters	Value
Size of experience replay buffer	10,000
Size of experience replay batch	128
Discounting factor	0.99
Maximum number of steps each episode	50
Learning rate of each neural network	0.0003

**Table 2:** The initial conditions of the three different cases.

	Target Trajectory	Target Velocity
Case 1	Square trajectory	0.5 m/s
Case 2	Polygonal trajectory	0.5 m/s
Case 3	Curve trajectory	0.5~1.2 m/s

## Data Availability

Not applicable.
